# Evaluating Student Satisfaction and Self-Confidence in Simulation-Based Anesthesiology Training among Final-Year Medical Students

**DOI:** 10.3390/healthcare12151521

**Published:** 2024-07-31

**Authors:** Abdullah Shbeer

**Affiliations:** Department of Surgery, College of Medicine, Jazan University, P.O. Box 114, Jazan 45142, Saudi Arabia; ashbeer@jazanu.edu.sa

**Keywords:** simulation-based training, medical education, student satisfaction, self-confidence, anesthesiology

## Abstract

Simulation-based training (SBT) is increasingly recognized in medical education for enhancing clinical skills and confidence. This study evaluated the impact of SBT on satisfaction and self-confidence among final-year medical students at Jazan University, Saudi Arabia. A total of 117 students participated in a cross-sectional study after attending SBT sessions focused on anesthesiology. Data were collected via a questionnaire assessing satisfaction and self-confidence. Statistical analyses included descriptive and inferential statistics. Results showed mean satisfaction and self-confidence scores of 3.64 ± 0.79 and 3.70 ± 0.90, respectively, with no significant differences by gender or age. A strong association was noted between the learning experience ratings and both satisfaction and self-confidence (*p* < 0.001). The highest satisfaction was with the instructor’s teaching style, whereas the lowest was with the motivation provided by teaching materials. For self-confidence, the highest scores related to the instructor’s guidance, while the lowest concerned the application of SBT to critical skills learning. A strong positive correlation was found between satisfaction and self-confidence (R = 0.847, *p* < 0.001). The study concludes that SBT significantly enhances student satisfaction and self-confidence, emphasizing the importance of learner-centered methods and interactive learning in medical education.

## 1. Introduction

Anesthesiology, a clinical specialty that originated over 150 years ago, has undergone a significant transformation, emerging as a complex and distinct medical field [1]. It focuses on administering anesthesia and managing pain during surgical interventions, demanding a high level of technical proficiency, in-depth knowledge, and sound clinical judgment [2]. This specialty involves using pharmacological agents to induce a controlled state of unconsciousness and analgesia, requiring a profound understanding of anatomy, physiology, and pharmacology. As such, anesthesiology plays a crucial role in modern medicine and healthcare [3]. The development of mannequin simulators and simulation programs for education and training in anesthesiology, which began over half a century ago with the introduction of the first fully automated cardiac auscultation mannequin, has led to significant advances and a permanent place for simulation-based education in anesthesiology and other medical and health educational programs [4]. 

The integration of simulation-based training (SBT) into anesthesiology education represents a significant advancement in preparing medical students and residents for the complexities and challenges inherent in anesthesia practice. Anesthesiology, a field characterized by high-stakes decision-making, rapid response to dynamic clinical situations, and the need for precision in procedural skills, presents an ideal context for the application of SBT methodologies. These simulation scenarios are designed to mimic a wide range of anesthetic situations, from routine inductions to critical emergencies, providing learners with the opportunity to practice technical skills, clinical judgment, and team-based communication in a safe, controlled environment [5,6]. A comprehensive and accurate understanding of anesthesia and the role of anesthetists is crucial for medical students, as it may motivate them to pursue this specialty in their future careers [7]. Moreover, possessing a thorough knowledge of the clinical aspects of anesthesia is imperative to ensure that individuals who qualify in this field are equipped to address patient queries and concerns related to anesthesia [8].

The efficacy of simulation in enhancing the educational outcomes of anesthesiology training is well-documented, with studies highlighting improvements in technical skill proficiency, decision-making accuracy, and crisis management abilities [9,10]. Furthermore, SBT offers a unique platform for assessing and enhancing non-technical skills, such as communication, teamwork, and leadership, which are critical to the successful practice of anesthesiology [11,12]. These aspects of SBT contribute to a holistic educational approach, aligning with the multifaceted competencies required in anesthesiology.

Given the critical role of self-confidence and satisfaction in the learning process, there is a growing interest in understanding how SBT influences these psychological constructs among anesthesiology students and residents. Self-confidence, defined as the belief in one’s ability to successfully execute a task or procedure, is particularly pertinent in the high-pressure environment of anesthesiology, where practitioners must rely on their skills and judgment under challenging circumstances [13]. Satisfaction with the learning experience is defined as the learners’ overall contentment and positive perception of the SBT experience in anesthesiology, encompassing aspects such as the effectiveness of teaching methods, suitability of learning materials, and enjoyment derived from the instructor’s teaching style. Evaluating satisfaction is crucial because it directly influences learners’ engagement, motivation, and perception of the training program’s educational value. High satisfaction levels can lead to increased enthusiasm, active participation, and commitment to learning. Moreover, assessing satisfaction provides valuable feedback to educators, enabling them to identify strengths and areas for improvement in the simulation-based curriculum, ultimately fostering a positive learning environment that promotes the acquisition of essential knowledge and skills in anesthesiology [14,15].

Despite the recognized benefits of SBT in anesthesiology education, studies specifically evaluating its impact on learners’ satisfaction and self-confidence are sparse, particularly among final-year medical students transitioning into residency programs. This study focuses on final-year medical students as they are at a pivotal phase in their medical education, necessitating the acquisition of technical and procedural skills, alongside the development of self-assurance and professional identity, as future anesthesiologists. Therefore, this study aims to evaluate the effects of SBT on the satisfaction and self-confidence of final-year medical students within an anesthesiology context. 

## 2. Materials and Methods

### 2.1. Study Design and Population

This cross-sectional study was conducted between January to December 2023, to assess the satisfaction and confidence of medical students in the College of Medicine at Jazan University who underwent SBT. The study population consisted of all 117 final-year medical students enrolled at Jazan University’s College of Medicine, Saudi Arabia. The questionnaire was administered immediately after the students completed the SBT sessions to capture their perceptions and experiences.

### 2.2. Ethical Considerations

The study was conducted in full compliance with the Declaration of Helsinki, and ethical approval was granted by the Institutional Review Board (IRB) of Jazan University. Participants were assured of their anonymity and confidentiality throughout the research process, with written informed consent obtained prior to participation. To protect participants’ anonymity and confidentiality, no personally identifiable information was collected.

### 2.3. Simulation-Based Training (SBT) Intervention

The SBT intervention was structured around core competencies in anesthesiology, administered over two months, with participants engaging in two-hour weekly sessions. The SBT included airway management, anesthesia induction, patient monitoring, and emergency response to anesthetic complications. The curriculum was designed in collaboration with experienced anesthesiologists and education specialists, incorporating high-fidelity manikins and state-of-the-art simulation technology.

Skills Lab Sessions: These sessions focused on developing procedural skills through the use of low to medium-fidelity simulators. Students practiced techniques in airway management, vascular access, and regional anesthesia under the supervision of faculty members.

Hands-on Simulation Exercises: High-fidelity simulation scenarios were employed to mimic real-life clinical situations, such as managing a difficult airway or responding to an intraoperative emergency. These sessions emphasized teamwork, decision-making, and the application of clinical knowledge in a simulated clinical environment.

### 2.4. Data Collection Tool

A self-administered structured questionnaire was utilized for data collection, comprising socio-demographic factors and a widely previously validated Students’ Satisfaction and Self-confidence Scale [16,17]. This scale has been used in multiple studies to assess student satisfaction and self-confidence in simulation-based learning environments [18,19]. The socio-demographic factors encompassed age, gender, and rating learning experience (ranging from poor to good). The Student Satisfaction and Self-Confidence in Learning scale comprises a 13-item instrument, designed to measure student satisfaction (five items) with the simulation activity and self-confidence in learning (eight items), using a five-point Likert-type scale ranging from strongly disagree to strongly agree. The questionnaire was administered in English and was made available to students through a unique barcode linked to a Google Form. Students were instructed to scan the barcode using their mobile phones to access the questionnaire. The average time students took to complete the questionnaire was approximately 13 min.

### 2.5. Data Analysis

The Statistical Package for Social Sciences (SPSS) version 26 software (IBM Corp., Armonk, NY, USA) was used to analyze the data. Descriptive statistics were used to evaluate the demographic data and means while frequency distributions were used to summarize the responses to the questionnaire. Moreover, non-parametric tests were used to analyze the data as the variables did not meet the assumptions of normality. The Mann-Whitney U Test and the Kruskal-Wallis one-way ANOVA were executed to find out the association between different variables. A Spearman correlation analysis was conducted to examine the relationship between student satisfaction and self-confidence. Statistical significance was set at a *p*-value of 0.05.

## 3. Results

### 3.1. Demographic Characteristics

The study included 117 final-year medical students, with a majority being female (61.54%), aged 24 years (40.17%), and those who rated their overall learning experience as “average” (47.86%) (Table 1). A significant association was observed between the rating of the learning experience and both satisfaction and self-confidence scores (*p* < 0.001), with students who rated their experience as “good” (44.44%) showing higher mean scores for satisfaction (4.13 ± 0.76) and self-confidence (4.23 ± 0.74) compared to those rating it as “average” or “poor”.

### 3.2. Student Satisfaction with Simulation-Based Training

The level of agreement and mean scores related to student satisfaction with the SBT are presented in Table 2. The mean scores for student satisfaction with SBT ranged from 3.60 to 3.67 across all items. The highest mean score was for the suitability of the instructor’s teaching style to students’ learning preferences (3.67 ± 0.97), followed closely by the effectiveness of teaching methods (3.66 ± 1.01) and the variety of learning materials and activities provided (3.66 ± 1.1). The enjoyment of the instructor’s teaching received a slightly lower score (3.62 ± 1.08). The lowest mean score was for the motivation provided by teaching materials (3.60 ± 1.1)

### 3.3. Self-Confidence in Learning

Table 3 illustrates the participants’ level of agreement related to self-confidence in learning. The mean scores for self-confidence in learning ranged from 3.56 to 3.91 across all items. The highest mean score was for the instructor’s responsibility to guide learning content (3.91 ± 1.12), followed by knowing how to seek help when concepts are unclear (3.79 ± 1.11) and the use of helpful resources by instructors (3.74 ± 1.06). Students also showed confidence in the coverage of critical content (3.71 ± 1.08) and developing skills for clinical tasks (3.69 ± 1.11). Lower mean scores were observed for mastering session content (3.56 ± 1.07) and using activities to learn critical aspects of skills (3.56 ± 1.11).

### 3.4. Overall Student Satisfaction and Self-Confidence in Learning

**Figure 1:**
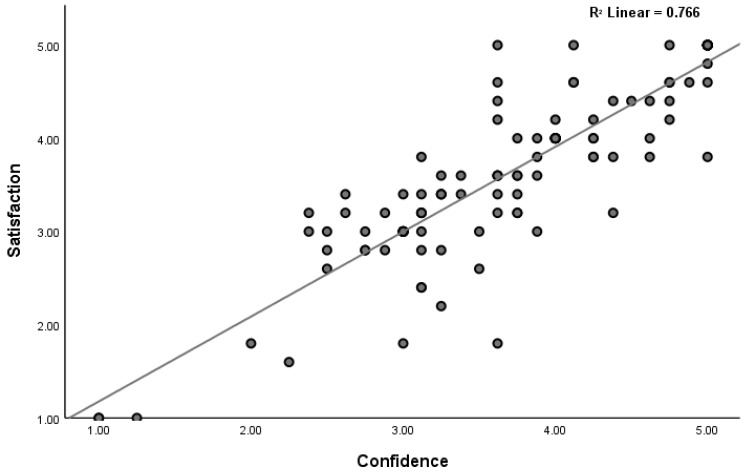
Correlation between satisfaction and self-confidence.

The overall mean score for Student Satisfaction was 3.64 ± 0.89, indicating a high level of satisfaction with the SBT. Similarly, the overall mean score for Self-Confidence in Learning was 3.70 ± 0.90, suggesting that students felt confident in their learning and skill development through the SBT. A strong and significant positive correlation was observed between satisfaction levels and self-confidence levels (*p* < 0.001) (Figure 1).

## 4. Discussion

The regular measurement of satisfaction and self-confidence among medical students is crucial for ensuring the effectiveness of SBT programs. Assessing these factors allows educators to identify areas for improvement and optimize the learning experience for students. The present study aimed to evaluate students’ satisfaction and self-confidence after attending SBT using skills labs and hands-on sessions, providing valuable insights into the impact of these educational interventions on final-year medical students in anesthesiology. 

The satisfaction with SBT in anesthesiology among final-year medical students, as indicated by mean scores ranging from 3.60 to 3.67 (the overall mean scores = 3.64 ± 0.89), reflects a generally positive reception, particularly regarding the suitability of the instructor’s teaching style (3.67 ± 0.97) and the effectiveness of teaching methods (3.66 ± 1.01). These findings are consistent with previous studies that highlight the importance of tailored teaching methods and diverse learning materials in enhancing student satisfaction [20]. For instance, a study on learner-centered, SBT in anesthesiology reported high satisfaction levels, with 95% of students finding the training conducive to learning operational skills and expressing overall satisfaction with the course [21]. This suggests that SBT is well-received in anesthesiology, particularly when it aligns with students’ learning preferences and employs effective teaching strategies. When comparing satisfaction with SBT in anesthesiology to other medical specialties, similar trends are observed. A meta-analysis of randomized controlled trials found that SBT generally leads to higher satisfaction and improved learning outcomes across various medical fields [3]. Additionally, a scoping review on the uses of simulation-based education for anesthesiology training, certification, and recertification highlighted its growing acceptance and effectiveness in critical care education [22]. These findings suggest that while there are specific areas for improvement, such as the motivation provided by teaching materials (3.60 ± 1.1), the overall satisfaction with SBT in anesthesiology is comparable to, if not slightly better than, other medical specialties, particularly when the training is well-structured and aligned with learner needs.

The self-confidence levels of students in anesthesiology after SBT show a generally positive trend, with mean scores ranging from 3.56 to 3.91 (the overall mean scores = 3.70 ± 0.90). The highest confidence was reported in the instructor’s guidance of learning content (3.91 ± 1.12), which is crucial for effective learning. This aligns with findings in other medical specialties where SBT has been shown to significantly enhance self-confidence [23]. This finding emphasizes the crucial role of instructors in guiding students’ learning and setting clear expectations for the SBT. Instructors who provide clear instructions, learning objectives, and feedback can help students to focus their learning efforts and build their self-confidence. Conversely, the item with the lowest mean score and agreement percentage was for students’ ability to effectively use SBT activities for skill development (3.56 ± 1.11, 49.57%). This indicates that some students may require additional support and guidance in understanding how to effectively utilize the SBT activities to develop their clinical skills. Providing students with explicit instructions, demonstrations, and opportunities for practice and feedback can help to bridge this gap and enhance their self-confidence in learning.

When examining the association between demographic variables and satisfaction/self-confidence scores, a significant association was observed between the rating of the learning experience and both satisfaction and self-confidence scores (*p* < 0.001). Students who rated their learning experience as “good” had higher mean scores compared to those who rated it as “average” or “poor”. This finding underlines the importance of creating positive learning experiences to maximize student satisfaction and self-confidence.

Furthermore, a strong positive correlation was observed between satisfaction and self-confidence (R = 0.847, *p* < 0.001), suggesting that as students’ satisfaction with the SBT increased, their self-confidence in learning also improved. This finding is consistent with previous studies that have shown that satisfaction levels positively impact self-confidence [24,25]. This relationship has been previously documented in the literature, highlighting the importance of creating engaging and satisfying learning experiences to foster self-confidence among students.

The findings of this study have significant practical implications for medical education, particularly in anesthesiology. The results demonstrate the effectiveness of incorporating SBT into the curriculum, leading to increased student satisfaction and self-confidence. Medical schools should integrate more SBT into their anesthesiology curricula, focusing on increasing the frequency and variety of hands-on sessions and skills labs. This approach creates a more engaging and rewarding educational environment, fostering increased student motivation, deeper learning, and the development of essential non-technical skills. To support effective SBT programs, institutions may need to invest in high-quality simulation equipment, dedicated spaces, and faculty development programs. Regular assessment of student satisfaction and self-confidence should be incorporated into program evaluations, providing valuable feedback for continuous refinement and improvement. The variability in student responses suggests a need for flexible learning approaches that cater to different learning styles within SBT programs. Ultimately, SBT offers a unique opportunity to bridge the gap between theoretical knowledge and clinical practice in anesthesiology education, potentially leading to better-prepared graduates entering residency programs.

### Limitations

This study has several limitations that should be considered when interpreting the results. Although the study utilized a previously validated Students’ Satisfaction and Self-confidence Scale, the assessment of student satisfaction was based on only five items, which may not fully capture the complexity of this concept in simulation-based anesthesiology training. The study was conducted with a single class of final-year medical students at one university, limiting the generalizability of the findings. The cross-sectional design provides only a snapshot of student satisfaction and self-confidence at a single point in time. The reliance on self-reported data may be subject to social desirability bias. While the study demonstrated a correlation between satisfaction and self-confidence, it cannot establish causality. These limitations suggest the need for future research with larger, more diverse samples, more comprehensive assessment tools, and longitudinal designs to further validate and expand upon the findings.

## 5. Conclusions

The present study demonstrates the positive impact of SBT on student satisfaction and self-confidence among final-year medical students in anesthesiology. The high overall mean scores and strong correlation between satisfaction and self-confidence underscore the effectiveness of the SBT program. The findings also emphasize the importance of well-designed teaching methods, skilled instructors, and positive learning experiences in fostering student satisfaction and self-confidence. Based on these results, it is recommended that medical schools continue to integrate SBT into their anesthesiology curricula and regularly assess student satisfaction and self-confidence to ensure the ongoing effectiveness of these educational interventions.

## Figures and Tables

**Table 1:** Demographic characteristics and mean scores of satisfaction and self-confidence among study participants.

Item	Variables	Count (%)	Satisfaction Mean ± SD	*p*-Value	Self Conf. Mean ± SD	*p*-Value
Gender	Female	72 (61.54%)	3.64 ± 0.95	0.89	3.67 ± 0.92	0.538
	Male	45 (38.46%)	3.64 ± 0.92		3.75 ± 0.87	
Age	23	41 (35.04%)	3.62 ± 0.97	0.86	3.72 ± 0.92	0.949
	24	47 (40.17%)	3.71 ± 0.80		3.75 ± 0.82	
	≥25	29 (24.79%)	3.56 ± 1.10		3.60 ± 1.01	
Rate your learning experience	Good	52 (44.44%)	4.13 ± 0.76	<0.001	4.23 ± 0.74	<0.001
	Average	56 (47.86%)	3.41 ± 0.73		3.44 ± 0.65	
	Poor	9 (7.69%)	2.18 ± 0.99		2.35 ± 1.00	

**Table 2:** Participants’ level of agreement related to Student Satisfaction.

Items	Disagreement	Neutral	Agreement	Mean ± SD
The teaching methods used in these courses (skills lab and hands-on sessions) were helpful and effective.	13 (11.11%)	33 (28.21%)	71 (60.68%)	3.66 ± 1.01
The skills lab and hands-on sessions provided me with a variety of learning materials and activities to promote my learning.	16 (13.68%)	31 (26.5%)	70 (59.83%)	3.66 ± 1.1
I enjoyed how my instructor taught the skills lab and hands-on sessions.	16 (13.68%)	34 (29.06%)	67 (57.27%)	3.62 ± 1.08
The teaching materials used in these skills lab and hands-on sessions were motivated and helped me to learn.	19 (16.24%)	32 (27.35%)	66 (56.41%)	3.60 ± 1.1
The way my instructor taught the skills lab and hands-on sessions was suitable to the way I learned.	10 (8.54%)	42 (35.9%)	65 (55.55%)	3.67 ± 0.97

**Table 3:** Participants’ level of agreement related to self-confidence.

Items	Disagreement	Neutral	Agreement	Mean ± SD
I am confident that I am mastering the content of the skills lab and hands-on sessions activity that my instructors presented to me.	16 (13.67%)	41 (35.04%)	60 (51.29%)	3.56 ± 1.07
I am confident that this skills lab and hands-on sessions covered critical content necessary for mastery of the curriculum.	16 (13.68%)	30 (25.64%)	71 (60.68%)	3.71 ± 1.08
I am confident that I am developing the skills and obtaining the required knowledge from this skills lab and hand-on sessions to perform necessary tasks in a clinical setting.	16 (13.68%)	30 (25.64%)	71 (60.68%)	3.69 ± 1.11
My instructors used helpful resources to teach the skills lab and hands-on sessions.	11 (9.40%)	34 (29.06%)	72 (61.54%)	3.74 ± 1.06
It is my responsibility as a student to learn what I need to know from this skills lab and hands-on session activity.	15 (12.82%)	34 (29.06%)	68 (58.12%)	3.68 ± 1.11
I know how to get help when I do not understand the concept covered in the skills lab and hands-on sessions.	12 (10.26%)	32 (27.35%)	73 (62.39%)	3.79 ± 1.11
I know how to use the skills lab and hands-on session activities to learn critical aspects of these skills.	19 (16.24%)	40 (34.19%)	58 (49.57%)	3.56 ± 1.11
It is the instructor’s responsibility to tell me what I need to learn from the skills lab and hands-on session activity content during class time.	12 (10.26%)	31 (26.5%)	74 (63.25%)	3.91 ± 1.12

## Data Availability

The data that support the findings of this study are available from the corresponding author upon reasonable request.
